# Changes in gray matter volume following electroconvulsive therapy in adolescent depression with suicidal ideation: A longitudinal structural magnetic resonance imaging study

**DOI:** 10.3389/fpsyt.2022.944520

**Published:** 2022-09-29

**Authors:** Xiao Li, Xiaolu Chen, Renqiang Yu, Linqi Dai, Ming Ai, Qian Huang, Yi Zhou, Wanjun Chen, Jiamei Guo, Anhai Zheng, Li Kuang

**Affiliations:** ^1^Department of Psychiatry, The First Affiliated Hospital of Chongqing Medical University, Chongqing, China; ^2^The First Branch, The First Affiliated Hospital of Chongqing Medical University, Chongqing, China; ^3^Department of Radiology, The First Affiliated Hospital of Chongqing Medical University, Chongqing, China

**Keywords:** MDD, ECT (electroconvulsive therapy), adolescent, Suicide Ideation, structural MRI (sMRI)

## Abstract

**Objective:**

We aimed to investigate changes in whole-brain gray matter volumes (GMVs) before and after electroconvulsive therapy (ECT) in adolescents with major depressive disorder (MDD) and suicidal ideation (SI).

**Methods:**

Thirty adolescents with MDD and SI were observed, and structural magnetic resonance imaging (sMRI) was performed at baseline and after ECT for each patient. But Twenty-five healthy controls (HCs) were scanned only at baseline. The voxel-based morphometry (VBM) techniques were used to examine GMVs.

**Results:**

Compared with HCs, MDDs at baseline showed decreased GMVs in the left middle temporal gyrus, right superior temporal gyrus, right middle temporal gyrus, left precuneus, right precuneus, and left superior frontal gyrus. After ECT, MDDs showed increased GMVs in the right superior frontal gyrus and right superior temporal gyrus. Pearson’s correlation found that Beck Scale for Suicide Ideation (BSSI) scores at baseline were negatively correlated with GMVs in the left superior frontal gyrus and HAMD and BSSI scores after ECT were negatively correlated with GMVs in the right superior temporal gyrus.

**Conclusion:**

Frontal–temporal–precuneus structure changes may be a potential cause of depressive and suicidal symptoms in adolescents. ECT may improve depressive and suicidal symptoms in adolescents by regulating brain structures to compensate original defects.

## Introduction

Suicide is the third leading cause of death in adolescents (1) and is associated with major depressive disorder (MDD) (2). In addition to suicide deaths, suicidal ideation (SI) and suicide attempts also warrant attention, the development of SI to SA is a distinct phenomenon with different explanations, such as depressive symptoms, hopelessness, or even impulsivity that can predict ideation, but they struggle to distinguish those who have SA from those who only have SI (3). Klonsky et al. found the combination of pain and hopelessness, especially when pain exceeds connectedness, was the main factor causing SI and dispositional, acquired, and practical contributors to increased capacity for suicide were the main factors causing progression from ideation to attempts (4). Globally, lifetime prevalence rates were approximately 9.2% for SI and 2.7% for SA (5), and the prevalence rate of SA in adolescents was 4.1% in the United States and 4.2% in Europe (6). SI is defined as “thoughts of death, dying, plans for suicide, or desire for death” (7, 8), and it is thought to be the first step leading to suicide (3). Tan et al. found 32.09% of 12,733 adolescents in China reported SI (9), and Liu et al found the rate of SI was 20.6% in adolescents in Shandong province in China (10). Additionally, MDD adolescents with SI always have worse clinical outcomes (11). Therefore, measuring SI in MDD adolescents is necessary and may help determine the risk of suicide.

Previous structural magnetic resonance imaging (sMRI) studies showed that abnormalities gray matter volumes (GMVs) were associated with suicide in mood disorders. For example, reduced GMVs in the left and right dorsolateral prefrontal cortex (DLPFC) and right ventral lateral prefrontal cortex (VLPFC) were detected in patients with MDD and SI compared with MDD patients without SI and HCs (12). A longitudinal follow-up study on adolescents found a reduction in dorsal striatal GMVs in MDD adolescents with SI and may predict SI (13). Generally, understanding of alterations in GMVs in MDD adolescents with SI is still limited but of necessity and significance.

Electroconvulsive therapy (ECT) is a quick method to reduce depressive and suicidal symptoms, but for MDD adolescents with SI, the treatment effect of ECT was not so good compared with that adults (14), and due to the side effect, the caregivers always do not accept ECT as the first treatment option, a survey of 7,469 Australian psychiatric patients who received ECT showed only 0.2% of them were younger than 18 years (15), and another study containing 12,608 participants who received ECT from China showed only 3.2% (406) of them were adolescents (16). However, there were still some studies that demonstrated ECT was a good treatment option for MDD adolescents (17, 18). Previous studies demonstrated ECT could alternate GMVs in MDD patients. Qiu et al. (19) found GMVs were increased in bilateral amygdala and hippocampus in MDDs after ECT. Nordanskog et al. (20) found increased GMVs in bilateral hippocampus after 6–12 ECT sessions in MDD patients, and the increased GMVs in bilateral hippocampus correlate with clinical symptoms. However, how ECT changes GMVs in MDD adolescents is still unclear.

To our knowledge, few studies have explored the changes in GMVs in MDD adolescents with SI after ECT; therefore, we have hypothesized that (1) the adolescents with MDD and SI will show changed GMVs compared with HCs and (2) ECT would make GMVs changed in MDD adolescents with SI.

## Materials and methods

### Subjects

Thirty adolescents with MDD and SI aged 12–17 years were recruited, and the diagnosis was confirmed by senior psychiatrists using the Mini International Neuropsychiatric Interview for Children and Adolescents (MINI-KID). The severity of symptoms was accessed by both 17-item Hamilton Depression Rating Scale (21) and the Beck Scale for Suicide Ideation (22) at baseline and after ECT. The Chinese version of HAMD and BSSI has been found to be reliable and valid (23, 24). The inclusion criteria were as follows: (1) patients who fulfilled the MDD diagnostic criteria of the Diagnostic and Statistical Manual of Mental Disorders, Fourth Edition; (2) patients with a total score of ≥ 17 points on the Hamilton Depression Rating Scale (HAMD-17); (3) first-onset MDD or diagnosed before but without antidepressants in recent 8 weeks, no history of ECT treatment; and (4) patients with suicidal ideation (SI) and Beck Scale for Suicide Ideation (BSSI) scores >11 points in recent one week. Patients were excluded if they had: (1) a neurological or serious physical condition, any history of alcohol or drug abuse, any other somatic diseases, or morphological anomalies of the brain; (2) any surgically placed electronic or metal materials that might interfere with MRI assessment; or (3) head motion exceeding 2.5 mm in translation or 2.5° in rotation.

Healthy controls were volunteers matched with the MDDs in sex, age, and educational level. The controls reported neither lifetime psychiatric disorder nor a family history of psychosis in their first-degree relatives. Otherwise, the exclusion criteria remained the same as MDDs.

The study protocol was approved by the Human Research and Ethics Committee of the First Affiliated Hospital of Chongqing Medical University (no. 2017-157). All adolescents and their caregivers gave written informed consent after being informed about the details of the study.

### Electroconvulsive therapy

The MDDs were treated by bi-temporal ECT using Thymatron DGx (Somatics, LLC, Lake Bluff, IL, USA) at the First Affiliated Hospital of Chongqing Medical University. The first three sessions of ECT took place on continuous days; the remaining sessions of ECT were performed every 2 days, with a break on weekends, and each patient would take eight times of ECT. We used low 0.25 mode, and the initial energy for ECT was determined according to the patient’s age: energy percent = age × 50%. The stimulation energy was adjusted based on the seizure time, and subsequent dosing was determined by seizure morphology adequacy. Anesthesia was induced with succinylcholine (0.5–1 mg/kg) and diprivan (1.5–2 mg/kg).

All MDDs received antidepressants during ECT sessions, with sertraline (*n* = 18, 60%), fluoxetine (*n* = 11, 36.7%), and escitalopram (*n* = 1, 0.03%). Twenty-six patients received antipsychotics, with quetiapine (*n* = 13, 43.3%), olanzapine (*n* = 8, 26.7%), aripiprazole (*n* = 4, 13.3%), and risperidone (*n* = 1, 0.03%). One patient received trihexyphenidyl (*n* = 1, 0.03%).

### Magnetic resonance imaging acquisition

MR images were obtained using a 3T GE Signa HDxt scanner (General Electric Healthcare, Chicago, IL, USA) with an eight-channel head coil. During scanning, subjects were told to keep their eyes closed and awake, not to focus their thoughts on anything in particular. Foam pads and earplugs were used to fix their heads to minimize head motion and reduce machine noise, respectively. The settings used to obtain the 3D T1-weighted anatomical images were as follows: a repetition time of 8.4 ms, a flip angle of 12°, an echo time of 3.3 ms, a field of view of 24 cm × 24 cm, a matrix of 256, a field of view of 24-cm slices, 158 axial slices, and a slice thickness of 1 mm.

### Voxel-based morphometry image preprocessing

The T1-weighted images were processed with Statistical Parametric Mapping 12 (SPM12) software package using voxel-based morphometry (VBM) toolbox. All T1 images were segmented into gray matter (GM), white matter (WM), and cerebrospinal fluid (CSF) using the “new segment” tool implemented in SPM12. During spatial normalization, inter-subject registration was achieved using respective registration based on group assignment. A modulation step was used to ensure that the overall amount of tissue in a class was unaltered. The segmented images were normalized to the Montreal Neurological Institute (MNI) template and were smoothed with an 8-mm full-width at half-maximum (FWHM) Gaussian filter. The voxel size of data acquisition was 1 mm^3^, and the voxel size of normalized data was 1.5 mm^3^.

### Statistical analysis

Demographic data and symptom scores were analyzed using SPSS (version 26.0; IBM Corp., Armonk, NY, USA). A two-sample *t*-test was performed in SPM12 to compare GMVs between MDDs at baseline and HCs, and paired *t*-tests were performed between MDDs pre- and post-ECT treatment. The GMV voxel was *p* < 0.001, the significance level was *p* < 0.05 with Gaussian random field (GRF) correction, and the cluster size was > 170.

At baseline, Pearson’s correlation analyses were performed to examine the correlations between GMVs in the brain regions with significant differences and clinical symptoms (HAMD/BSSI). After ECT, Pearson’s correlation analyses were performed to examine whether the changes in these measures were correlated with the changes in clinical symptoms.

## Results

### Clinical outcomes

The psychological measurements and demographic data are listed in Table 1. There were no significant differences between MDDs and HCs in age, sex, and educational level (*p* > 0.05). After ECT sessions, the total scores on the HAMD and BSSI were significantly decreased (*p* < 0.05) (Table 2).

**TABLE 1:** Demographics and clinical characteristics between HCs and MDDs.

Characteristics	HC (*n* = 25)	MDD (*n* = 30)	*P*-value
Age, mean (SD), y	15.48 (1.87)	14.60 (1.45)	0.197*
Sex (male/female)	6/19	8/22	0.657#
Duration time of symptoms, mean (SD), y	–	1.1 (1.0)	–
Education years, mean (SD), y	9.68 (2.21)	8.50 (1.70)	0.143*
HAMD, mean (SD)	1.60 (2.06)	29.03 (6.02)	<0.001*
BSSI, mean (SD)	0	22.10 (5.73)	<0.001*

MDD, major depressive disorder; HC, healthy control; HAMD, Hamilton Depression Scale; BSSI, Beck Scale for Suicide Ideation; SD, standard deviation.

*Two-sample *t*-test.

^#^Chi-square test.

**TABLE 2:** Comparison of the severity of clinical symptoms between pre- and post-ECT.

Characteristic	Pre-ECT	Post-ECT	*P*
HAMD, mean (SD)	29.03 (6.02)	13.77 (8.89)	<0.001*
BSSI, mean (SD)	22.10 (5.73)	8.10 (6.94)	<0.001*

ECT, electroconvulsive therapy; HAMD, Hamilton Depression Scale; BSSI, Beck Scale for Suicide Ideation; SD, standard deviation.

*Paired *t*-test.

### Neuroimaging comparisons between major depressive disorders at baseline and healthy controls

**FIGURE 1:**
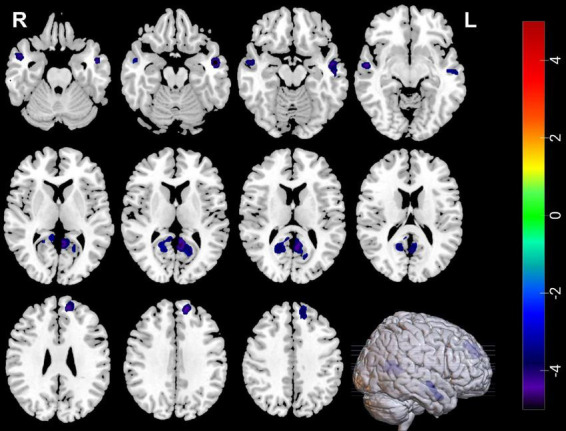
Blue regions mean decreased GMVs in the left middle temporal gyrus, right superior temporal gyrus, right middle temporal gyrus, left precuneus, right precuneus, and left superior frontal gyrus of MDDs compared with HCs (GMV voxel was *p* < 0.001; the significance level was *p* < 0.05 with GRF correction).

**FIGURE 2:**
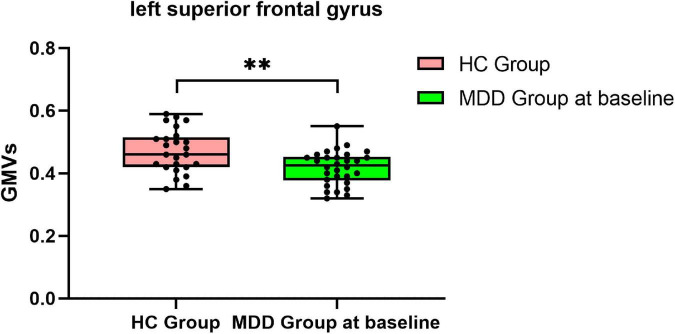
Bar graph showing decreased GMVs in the left superior frontal gyrus in MDDs compared with HCs, ^**^p < 0.01.

Compared with HCs, MDDs showed decreased GMVs in the left middle temporal gyrus, right superior temporal gyrus, right middle temporal gyrus, left precuneus, right precuneus, and left superior frontal gyrus (*p* < 0.05 with GRF correction) (Figure 1 and Table 3) and the bar graph showed decreased GMVs in the left superior frontal gyrus in MDDs (*p* < 0.01) (Figure 2).

**TABLE 3:** Significant differences in GMVs between MDDs and HCs.

Brain regions	L/R	Voxel size	Peak *T*-value		MNI coordinates	
Middle Temporal gyrus	R	231	−4.7436	48	4.5	−27
Superior Temporal gyrus	R	177	−4.6108	56	−6	−11
Middle Temporal gyrus	L	608	−5.3776	−51	−3	−19.5
Precuneus	L	941	−4.8458	−6	−52.5	9
Precuneus	R	579	−4.2185	9	−43.5	10.5
Superior frontal gyrus	L	699	−4.7427	−12	48	28.5

GMV, gray matter volume; MNI, Montreal Neurological Institute.

### Neuroimaging comparisons between pre- and post-electroconvulsive therapy

**FIGURE 3:**
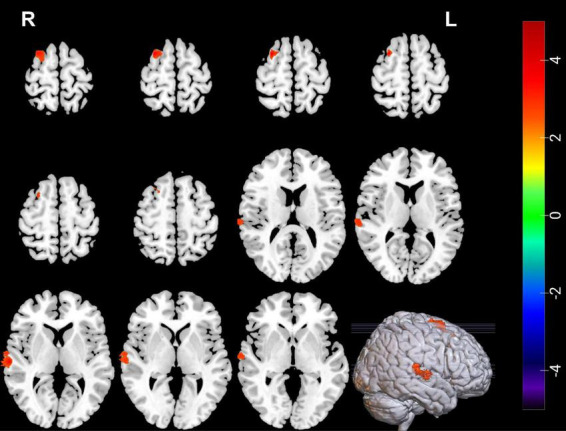
Red regions mean increased GMVs in the right superior temporal gyrus and right superior frontal gyrus of MDDs after ECT (GMV voxel was *p* < 0.001; the significance level was *p* < 0.05 with GRF correction).

**FIGURE 4:**
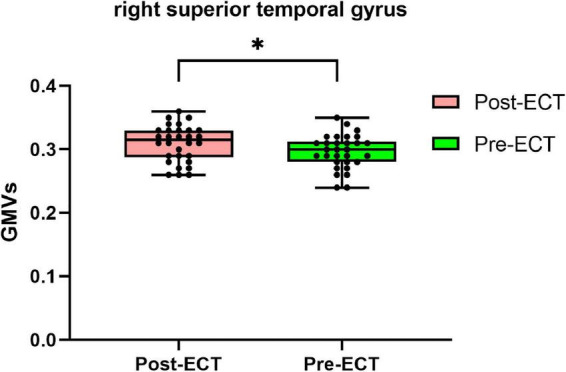
Bar graph showing increased GMVs in the right superior temporal gyrus in MDDs after ECT, *p < 0.05.

Compared with pre-ECT, patients showed increased GMVs in the right superior frontal gyrus and right superior temporal gyrus (*p* < 0.05 with GRF correction) (Figure 3 and Table 4) and the bar graph showed increased GMVs in the right superior temporal gyrus in MDDs after ECT (*p* < 0.05) (Figure 4).

**TABLE 4:** Significant differences in GMVs in MDDs between pre- and post-ECT.

Brain regions	L/R	Voxel size	Peak *T*-value		MNI coordinates	
Superior Temporal gyrus	R	608	3.7196	61.5	−18	4.5
Superior Frontal gyrus	R	425	3.8632	27	9	63

GMV, gray matter volume; MNI, Montreal Neurological Institute.

### Pearson’s correlation analysis

**FIGURE 5:**
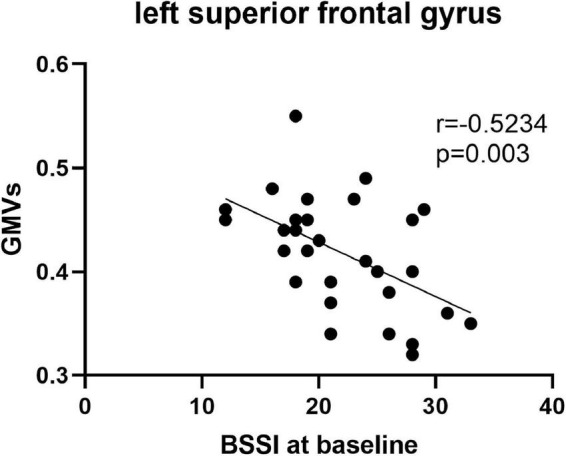
Negative correlations between the BSSI at baseline and GMVs in the left superior frontal gyrus (*r* = –0.5234, *p* = 0.003).

**FIGURE 6:**
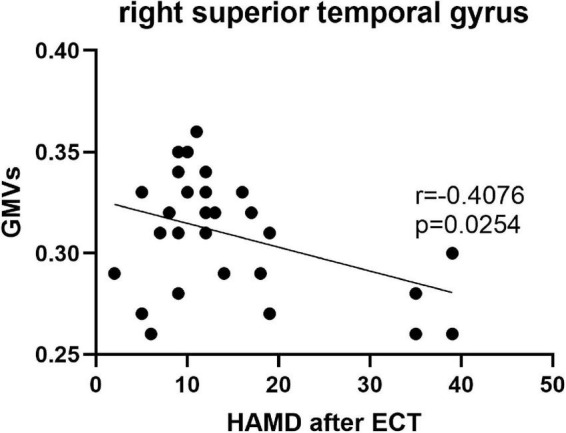
Negative correlations between the HAMD after ECT and GMVs in the right superior temporal gyrus (*r* = –0.4076, *p* = 0.0254).

**FIGURE 7:**
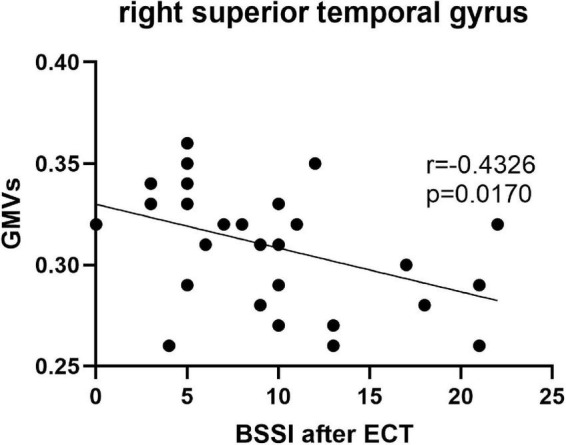
Negative correlations between the BSSI after ECT and GMVs in the right superior temporal gyrus (*r* = –0.4326, *p* = 0.0170).

Pearson’s correlation found that the BSSI scores at baseline were negatively correlated with GMVs in the left superior frontal gyrus (*r* = −0.5234, *p* = 0.003) (Figure 5), the HAMD scores after ECT were negatively correlated with GMVs in the right superior temporal gyrus (*r* = −0.4076, *p* = 0.0254) (Figure 6), and the BSSI scores after ECT were negatively correlated with GMVs in the right superior temporal gyrus (*r* = −0.4326, *p* = 0.0170) (Figure 7). There was no correlation between changed HAMD/BSSI scores and GMVs.

## Discussion

This study found the HAMD score and BSSI score of MDD adolescents with SI significantly reduced after ECT, indicating that ECT could quickly decrease the depressive and suicidal symptoms of adolescent patients. In our study, we used VBM techniques and found decreased GMVs in the left middle temporal gyrus, right superior temporal gyrus, right middle temporal gyrus, left precuneus, right precuneus, and left superior frontal gyrus compared with HCs and increased GMVs in the right superior frontal gyrus and right superior temporal gyrus after ECT.

The frontal lobe locates in the front of the brain and is one of the most important areas, which is closely related to the individual’s emotional regulation, thinking process, attention function, problem-solving, demand motivation, behavior planning, and other advanced cognitive activities. In this study, GMVs in the superior frontal gyrus changed in patients at baseline compared with HCs and also showed in MDDs before and after ECT, indicating that the superior frontal gyrus was an important area for the onset of depression and might be the area for the ECT response. Ma et al. found decreased GMV in the medial part of the left superior frontal gyrus in patients with depressive symptoms (25), which was consistent with our results. Seok et al. found decreased GMV in the right superior frontal gyrus in adolescents with irritability compared with healthy youths (26). Interestingly, we found ECT increased GMV in the right superior frontal gyrus, but not in the left superior frontal gyrus, and this inconsistency of two comparison results may be related to that ECT could trigger both efficacy and side effect. The precuneus is involved in many high-level cognitive functions of the brain, including episodic memory, self-attention, and information-related processing. Therefore, changes in brain structure in the region reflect abnormalities in emotional control, thought processes, and negative behaviors in patients with depression and suicide. In the current study, GMVs in precuneus in the MDDs at baseline significantly reduced, suggesting the abnormality of precuneus might be related to symptom onset of MDD adolescents with SI.

The middle temporal gyrus is involved in cue-directed attention and working memory (27, 28). Previous studies showed MDD patients had reduced GMVs in the temporal cortex (29, 30). In this study, GMVs in bilateral middle temporal gyrus of the patient group at baseline reduced compared with HCs, which might reflect depressive symptoms. Previous studies found that ECT increased GMVs in the temporal cortex in patients with depression (31, 32). In addition to the middle temporal gyrus, the right superior temporal gyrus is also an important brain region for emotion processing. Previous studies showed that the superior temporal gyrus and middle temporal gyrus were related not only to recall of personal experiences, but also to perception of intentional behavior and memory (33, 34). In addition, these two brain regions were also involved in the regulation of emotional information and cognition (35–37). Therefore, aberrations of GMV in these two regions may lead to abnormal spontaneous brain activity and might contribute to emotional dysregulation, which would increase the risk of suicide in depression. Soloff et al. (38) found that the superior/middle temporal gyrus was negatively correlated with the impulsivity of the low-fatality suicide attempters, but positively correlated with the aggressiveness of the high-fatality suicide attempters. Therefore, suicide attempts might be related to impulsivity personality traits mediated by the superior/middle temporal gyrus.

In addition to sMRI, fMRI also focused on the relationship between the superior temporal gyrus and suicide. A previous study (39) showed the ALFF activity in the right superior temporal gyrus enhanced significantly in depressed patients with SA, but no similar results were found in the comparison between depressed patients without SA and HCs. Van Heeringen et al. (40) also found increased blood perfusion in the temporal lobe of MDD patients. As the superior temporal gyrus is involved in the regulation of emotion and cognition, researchers believe that the abnormal ALFF activity in depressed patients with suicide may lead to suicidal behavior through weakened decision making and increased impulsivity. In addition, studies showed that the right superior temporal gyrus was activated in healthy people when performing mind tasks. Suicide attempters were considered to have impaired cognitive control and decision-making ability, and cognitive rigidity and impaired decision-making ability were particularly pronounced in high-fatality suicide attempters.

We found ECT changed GMVs in the right superior temporal gyrus, and previous studies paid more attention to alteration in the brain function in this area after ECT. Wang et al. found the changes in local FCD of the right superior temporal gyrus were significantly correlated with the changes in Hamilton Rating Scale for Depression (HRSD) scores in MDD patients before and after ECT (41). Previous studies also focused on the superior temporal gyrus changed in MDD adolescents with suicidal behaviors. Pan et al. (42) found GMVs in the right superior temporal gyrus reduced in MDD adolescents with a history of suicidal symptoms, and abnormal temporal–parietal GMVs were associated with an increased risk of suicide in youth with depression (43). So, we considered ECT could decrease the depressive and suicide symptoms by regulating the right superior temporal gyrus of brain.

Several limitations of our current study should be noted. First, our sample size was small, and further studies with larger samples are needed to verity our findings. Second, we did not assess childhood trauma or maltreatment in the MDDs and HCs, which could influence the brain structure. Third, we did not evaluate the side effect caused by ECT. Fourth, patients received antidepressants during ECT sessions, and though the duration of medications is only 2 weeks and antidepressants were always thought not to work (44, 45), we still could not exclude the impact on the brain structure.

## Conclusion

Frontal–temporal–precuneus structural changes may be a potential cause of depressive and suicidal symptoms in adolescents. ECT may improve depressive and suicidal symptoms in adolescents by regulating brain structures to compensate original defects.

## Data availability statement

The raw data supporting the conclusions of this article will be made available by the authors, without undue reservation.

## Ethics statement

The studies involving human participants were reviewed and approved by the Human Research and Ethics Committee of The First Affiliated Hospital of Chongqing Medical University. Written informed consent to participate in this study was provided by the participants’ legal guardian/next of kin.

## Author contributions

XL conceived the structure of the manuscript and wrote the manuscript. RY, YZ, QH, and LD prepared the samples and performed the fMRI. XC analyzed the data. JG, AZ, and WC performed the ECT in patients. MA and LK critically reviewed the manuscript. All authors have read and approved the final manuscript.
